# Antihypertensive Behandlung für Nierentransplantatempfänger

**DOI:** 10.1007/s00120-025-02553-1

**Published:** 2025-03-05

**Authors:** Laila Schneidewind

**Affiliations:** 1 Universitätsklinik für Urologie, Inselspital Bern, Freiburgstr. 37, 3010 Bern, Schweiz; 2 UroEvidence@Deutsche Gesellschaft für Urologie, Berlin, Deutschland

## Originalpublikation

Natale P, Mooi PKL, Green SC, Cross NB, Cooper TE, Webster AC, Masson P, Craig JC, Strippoli GFM (2024) Antihypertensive treatment for kidney transplant recipients. Cochrane Database Syst Rev. 7: CD003598. 10.1002/14651858.CD003598.pub3.

## Übersetzung

### Hintergrund.

Die vergleichenden Wirkungen spezifischer blutdrucksenkender Behandlungen auf patientenrelevante Endpunkte nach einer Nierentransplantation sind ungewiss. Unser Cochrane Review von 2009 ergab, dass Kalziumkanalblocker (CCB) die Transplantatfunktion verbessern und den Transplantatverlust verhindern, während die Evidenz für andere blutdrucksenkende Behandlungen begrenzt war. Dies ist eine Aktualisierung des Cochrane Reviews von 2009.

### Ziele.

Es wird der Nutzen und der Schaden verschiedener Klassen und Kombinationen blutdrucksenkender Medikamente bei nierentransplantierten Patienten verglichen.

### Suchmethodik.

Wir kontaktierten den Informationsspezialisten und durchsuchten das Cochrane Kidney and Transplant Register of Studies bis zum 3. Juli 2024 mit Suchbegriffen, die für diese Übersichtsarbeit relevant sind. Die im Register enthaltenen Studien wurden durch Recherchen in CENTRAL, MEDLINE, EMBASE, Konferenzberichten, dem Suchportal der International Clinical Trials Registry Platform (ICTRP) und ClinicalTrials.gov ermittelt.

### Auswahlkriterien.

In Frage kamen randomisierte kontrollierte Studien (RCT) und Quasi-RCT, in denen ein beliebiger blutdrucksenkender Wirkstoff bei Empfängern eines funktionierenden Nierentransplantats für mindestens 2 Wochen lang untersucht wurde.

### Datensammlung und Datenanalyse.

Zwei Autoren bewerteten unabhängig voneinander die Risiken einer Verzerrung und extrahierten die Daten. Die Behandlungsschätzungen wurden unter Verwendung des Random-effekts-Modells zusammengefasst und als relatives Risiko (RR) oder mittlere Differenz (MD) mit 95 %-Konfidenzintervallen (KI) ausgedrückt. Die Sicherheit der Evidenz wurde anhand des GRADE-Verfahrens („grades of recommendation, assessment, development and evaluation“) bewertet. Zu den primären Endpunkten zählten Todesfälle insgesamt, Transplantatverlust und Nierenfunktion.

### Hauptergebnisse.

Siebenundneunzig Studien (8706 Teilnehmende) wurden einbezogen. In einer Studie wurde die Behandlung bei Kindern untersucht. Das Gesamtrisiko der Verzerrung war in allen Bereichen unklar bis hoch.

Im Vergleich zu Placebo oder der Regelversorgung allein verringern CCB wahrscheinlich die Gesamttodesfälle (23 Studien; 3327 Teilnehmende: RR 0,83; 95 %-KI 0,72 bis 0,95; I^2^ = 0 %; Evidenz von moderater Vertrauenswürdigkeit) und Transplantatverlust (24 Studien; 3577 Teilnehmende: RR 0,84; 95 %-KI 0,75 bis 0,95; I^2^ = 0 %; Evidenz von moderater Vertrauenswürdigkeit). CCB können die geschätzte glomeruläre Filtrationsrate (eGFR) wenig oder gar nicht beeinflussen (11 Studien; 2250 Teilnehmende: MD 1,89 ml/min/1,73 m^2^; 95 %-KI −0,70 bis 4,48; I^2^ = 48 %; Evidenz von niedriger Vertrauenswürdigkeit) und akuter Abstoßung (13 Studien; 906 Teilnehmende: RR 10,8; 95 %-KI 0,85 bis 1,35; I^2^ = 0 %; Evidenz von moderater Vertrauenswürdigkeit). CCB können den systolischen Blutdruck (SBP) senken (3 Studien; 329 Teilnehmende: MD −5,83 mm Hg; 95 %-KI −10,24 bis −1,42; I^2^ = 13 %; Evidenz von niedriger Vertrauenswürdigkeit) und diastolischer Blutdruck (DBP; 3 Studien, 329 Teilnehmende: MD −3,98 mm Hg, 95 % KI −5,98 bis −1,99; I^2^ = 0 %; Evidenz von niedriger Vertrauenswürdigkeit). Die Wirkungen von CCB auf die Proteinurie sind ungewiss.

Im Vergleich zu Placebo oder der Regelversorgung allein machen Angiotensin-converting-Enzymhemmer (ACEi) möglicherweise nur einen geringen oder gar keinen Unterschied bei der Gesamtsterblichkeit (7 Studien; 702 Teilnehmende: RR 1,13; 95 %-KI 0,58 bis 2,21; I^2^ = 0 %; Evidenz von niedriger Vertrauenswürdigkeit), Transplantatverlust (6 Studien, 718 Teilnehmende: RR 0,75; 95 %-KI 0,49 bis 1,13; I^2^ = 0 %; Evidenz von niedriger Vertrauenswürdigkeit), eGFR (4 Studien; 509 Teilnehmende: MD −2,46 ml/min/1,73 m^2^; 95 %-KI −7,66 bis 2,73; I^2^ = 64 %; Evidenz von niedriger Vertrauenswürdigkeit) und akute Abstoßung (4 Studien; 388 Teilnehmende: RR 1,75; 95 %-KI 0,76 bis 4,04; I^2^ = 0 %; Evidenz von niedriger Vertrauenswürdigkeit). ACEi können die Proteinurie verringern (5 Studien; 441 Teilnehmende: MD −0,33 g/24 h; 95 %-KI −0,64 bis −0,01; I^2^ = 67 %; Evidenz von niedriger Vertrauenswürdigkeit), hatte aber unsichere Wirkungen auf SBP und DBP.

Im Vergleich zu Placebo oder der Regelversorgung allein machen Angiotensinrezeptorblocker (ARB) möglicherweise nur einen geringen oder gar keinen Unterschied bei der Gesamtsterblichkeit (6 Studien; 1041 Teilnehmende: RR 0,69; 95 %-KI 0,36 bis 1,31; I^2^ = 0 %; Evidenz von niedriger Vertrauenswürdigkeit), eGRF (5 Studien; 300 Teilnehmende: MD −1,91 ml/min/1,73 m^2^; 95 %-KI −6,20 bis 2,38; I^2^ = 57 %; Evidenz von niedriger Vertrauenswürdigkeit) und akute Abstoßung (4 Studien, 323 Teilnehmende: RR 1,00; 95 %-KI 0,44 bis 2,29; I^2^ = 0 %; Evidenz von niedriger Vertrauenswürdigkeit). ARB können den Transplantatverlust verringern (6 Studien; 892 Teilnehmende: RR 0,35; 95 %-KI 0,15 bis 0,84; I^2^ = 0 %; Evidenz von niedriger Vertrauenswürdigkeit), SBP (10 Studien; 1239 Teilnehmende: MD −3,73 mm Hg; 95 %-KI −7,02 bis −0,44; I^2^ = 63 %; Evidenz von moderater Vertrauenswürdigkeit) und DBP (9 Studien; 1086 Teilnehmende: MD −2,75 mm Hg; 95 %-KI −4,32 bis −1,18; I^2^ = 47 %; Evidenz von moderater Vertrauenswürdigkeit), hat aber ungewisse Wirkungen auf die Proteinurie.

Die Wirkungen von CCB, ACEi oder ARB im Vergleich zu Placebo oder der Regelversorgung allein auf kardiovaskuläre Endpunkte (einschließlich tödlicher oder nicht-tödlicher Myokardinfarkte, tödlicher oder nicht-tödlicher Schlaganfälle) oder andere unerwünschte Ereignisse waren ungewiss.

Die Wirkungen von ACEi plus ARB-Doppeltherapie, Alpha-Blockern und Mineralokortikoidrezeptorantagonisten im Vergleich zu Placebo oder alleiniger Regelversorgung wurden nur selten untersucht.

Es gab kaum direkte Vergleiche zwischen ACEi, ARB oder Thiazid und CCB, ACEi und ARB, CCB oder ACEi und Alpha- oder Beta-Blockern oder ACEi plus CCB-Doppeltherapie und ACEi- oder CCB-Monotherapie. In keiner der Studien wurden Ergebnisse zur Krebserkrankung oder zur Teilhabe am Leben (z. B. Rückkehr zur Arbeit, soziale Aktivitäten, Selbständigkeit) berichtet.

### Schlussfolgerung der Autoren.

Bei Nierentransplantatempfängern führt eine CCB-Therapie zur Senkung des Blutdrucks im Vergleich zu Placebo oder Regelversorgung allein wahrscheinlich zu weniger Todesfällen und Transplantatverlusten, während ARB den Transplantatverlust verringern kann. Die Wirkungen von ACEi und ARB im Vergleich zu Placebo oder der Regelversorgung auf andere patientenzentrierte Endpunkte waren ungewiss. Die Wirkungen der dualen Therapie, der Alpha-Blocker und der Mineralokortikoidrezeptorantagonisten im Vergleich zu Placebo oder der alleinigen Regelversorgung sowie die vergleichenden Wirkungen der verschiedenen Behandlungen waren ungewiss.

## Kommentar

Bei dem vorliegenden aktuellen Cochrane Review handelt es sich auch für Urologen um eine wichtige Arbeit – selbst wenn das Thema zunächst vorrangig nephrologisch anmutet. Denn das kontrovers diskutierte Organspendegesetz und die langen Wartezeiten auf eine Nierentransplantation in Deutschland machen es noch essenzieller, die Ergebnisse in jeglicher Hinsicht weiter zu verbessern, um das Vertrauen in der Bevölkerung für dieses Verfahren zu stärke [1]. Weiterhin betrifft Bluthochdruck 50–90 % der Empfänger von Nierentransplantaten [2, 3]. Die meisten Patienten haben bereits vor der Transplantation einen Bluthochdruck. Dabei ist Bluthochdruck ein wichtiger Risikofaktor für atherosklerotische kardiovaskuläre Erkrankungen und wird mit vorzeitigem Tod von Patienten auf der Warteliste ebenso assoziiert wie der frühzeitige Transplantatverlust nach erfolgter Transplantation [2, 4]. Zusätzlich wird Bluthochdruck nach einer Transplantation oft durch immunsuppressive Medikamente, Gewichtszunahme und Transplantatdysfunktion verschlechtert und ist bei bis zu 19 % der Nierentransplantierten behandlungsresistent [2, 5]. Damit ist eine konsequente Blutdruckeinstellung bzw. Überwachung für die Langzeitergebnisse der Nierentransplantation unabdinglich und so sollten auch Urologen über Grundkenntnisse in diesem Bereich verfügen.

Bei dem vorliegenden Cochrane Review handelt es sich um eine sehr aktuelle Arbeit mit der letzten Suche im Juli 2024. Weiterhin war das Update des vorherigen Cochrane Review unbedingt erforderlich, da seit 2009 v. a. Studien mit ACEi hinzugekommen sind. Interessanterweise hat der Beitrag zwei Zielstellungen: zum einen den Vergleich des relativen Nutzens und Schadens von verschiedenen Klassen oder Kombinationen von blutdrucksenkenden Arzneimitteln bei Empfängern von Nierentransplantaten und zum anderen die Bewertung von Unterschieden in den Effekten je nach Studie, Intervention und Patientencharakteristika, einschließlich der Unterschiede im erreichten Blutdruck. Hervorzuheben ist außerdem die sehr gute Methodik dieser Übersichtsarbeit inklusive detaillierter Subgruppenanalysen.

Die Aktualität des vorliegenden Cochrane Reviews wird unterstrichen durch die Tatsache, dass in einer am 29. Dezember 2024 durchgeführten Update-Suche keine weiteren Studien mit den entsprechenden Einschlusskriterien identifiziert werden konnten. Selbst die in der Übersichtsarbeit erwähnte laufende Untersuchung ist bisher nicht publiziert worden [6]. Allerdings ist kritisch anzumerken, dass die inkludierten Studien teils erhebliche methodische Probleme aufweisen. Teilweise handelt es sich um Untersuchungen mit sehr kleinen Fallzahlen oder unterpowerte Studien. Zusätzlich werden nur kurze Beobachtungszeiträume betrachtet und unterschiedliche Definitionen, auch von Outcome-Parametern, verwendet.

Konsequenterweise muss ebenfalls angemerkt werden, dass einige wichtige Aspekte der Nierentransplantation aufgrund der methodischen Schwächen der inkludierten Studien nur unzulänglich adressiert werden. Lediglich eine eingeschlossene Studie beschäftigt sich mit der pädiatrischen Nierentransplantation. Allerdings wären auch Untersuchungen bei Kindern- und Jugendlichen absolut erstrebenswert, um die langzeitige Transplantatfunktion zu gewährleisten. Weiterhin ist gerade hier der Vergleich mit erwachsenen Empfängern einer Nierentransplantation nur sehr eingeschränkt möglich, da bei Kindern andere pharmakokinetische und -dynamische Eigenschaften/Aspekte zu erwarten sind [7]. Unglücklicherweise gibt es hier eine erhebliche Evidenzlücke, die unbedingt geschlossen werden muss. Weitere offene Aspekte sind Kombinationstransplantationen, z. B. bei Niere- und Pankreastransplantationen, sowie der Langzeitverlauf nach der Transplantation und der Einfluss auf die verschiedenen Organfunktionen, wie der Entgiftungsfunktion.

Für die klinische Praxis sehen die Autoren des vorliegenden Cochrane Reviews folgende Implikationen: CCB reduzieren wahrscheinlich die Todesfälle insgesamt und den Transplantatverlust, während ARB den Transplantatverlust im Vergleich zu Placebo oder einer Kontrollgruppe reduzieren.

Die Wirksamkeit einer ACEi- oder ARB-Monotherapie auf Gesamtüberleben, kardiovaskuläre (wie z. B. Myokardinfarkte) und nierenbedingte (z. B. eine humorale Abstoßung) Nierenereignisse im Vergleich zu Placebo oder der Standardbehandlung war ungewiss. Die Wirkungen der dualen Therapie, der Alpha-Blocker und der Mineralokortikoidrezeptorantagonisten (MRA) im Vergleich zu Placebo oder keiner Behandlung und in einem direkten Vergleich waren ungewiss. Die absoluten Wirkungen von CCB im Vergleich zu Placebo zeigten, dass CCB wahrscheinlich 28 Patienten vor dem Tod bewahrten sowie 34 Patienten vor einem Transplantatverlust für je 1000 Patienten, die ein Jahr lang behandelt worden sind. Die absoluten Wirkungen von ACEi oder ARB im Vergleich zu Placebo waren niedriger, wahrscheinlich aufgrund von Äraunterschieden zwischen den beiden Gruppen. Da sich jedoch der Behandlungsstandard für die Immunsuppression von Cyclosporin-A-basierten Therapien auf Tacrolismus-basierte Therapien verlagert, könnte die Anwendbarkeit dieser Erkenntnisse eingeschränkt sein. Es gab nur wenige Belege für Unterschiede zwischen ACEi oder ARB im Vergleich zu CCB sowie begrenzte Daten darüber, ob eine Untergruppe von Patienten von ACEi oder ARB profitieren könnte und nur wenige Daten über den relativen Nutzen anderer blutdrucksenkender Behandlungen bei nierentransplantierten Patienten, die als Grundlage für die klinische Praxis dienen könnten. Unerwünschte Ereignisse wurden nur selten berichtet, und nur eine Studie wurde an Kindern durchgeführt [2]. Zusammenfassend ist also zu bemerken, dass es aktuell die beste Evidenz für den Einsatz von CCB in der Blutdruckeinstellung Nierentransplantierter gibt, allerdings werden dringend aktuelle Daten, insbesondere für ACEi unter Standardimmunsuppression, benötigt.

Diese Implikationen für die klinische Praxis machen bereits stringent auf die Evidenzlücken aufmerksam und führen zu den Implikationen für die weitere Forschung. So sind künftige RCT mit ausreichender Stichprobengröße und längerer Nachbeobachtung erforderlich, um den Nutzen und Schaden von CCB oder anderen blutdrucksenkenden Behandlungen bei Patienten, die eine Nierentransplantation benötigen, zu bewerten. Weitere Forschungsarbeiten werden wahrscheinlich die geschätzten Auswirkungen von Behandlungen bei Nierentransplantatempfängern verändern. So gibt es z. B. nur wenige Daten aus direkten Studien zwischen CCB und Renin-Angiotensin-System(RAS)-Blockern. Zusätzlich gibt es nur unzureichende Daten, um zu wissen, ob es signifikante Unterschiede zwischen den verschiedenen RAS-Blockern bestehen. Es sind außerdem weitere Studien mit direkten Vergleichen zwischen zwei verschiedenen Klassen von blutdrucksenkenden Behandlungen erforderlich. Die wichtigsten, für die Patienten relevanten Ergebnisse (einschließlich Tod, Herz-Kreislauf-Erkrankungen, Transplantatgesundheit, Auftreten von Krebserkrankungen, Infektionen, Teilhabe am Leben) sollten zur Unterstützung der klinischen Praxis angegeben werden. Eine Bewertung der Kosteneffektivität von Behandlungen zur Senkung des Blutdrucks bei Menschen, die eine Nierentransplantation benötigen, würde die Entscheidungsfindung von Gesundheitsdienstleistern und politischen Entscheidungsträgern unterstützen [2].

Zusammenfassend ist dringend weitere Forschungsarbeit mit robusten RCT in diesem Bereich notwendig, um die Ergebnisse der Nierentransplantation weiter zu verbessern und das Vertrauen in dieses Verfahren in der Bevölkerung zu stärken. Das vorliegende Cochrane Review liefert dabei vor allem wichtige Aspekte für die stringente Studienplanung.

## Fazit für die Praxis

- Eine konsequente Blutdruckeinstellung für die Langzeitergebnisse der Nierentransplantation ist unabdinglich.
- Eine Kalziumkanalblocker (CCB)-Therapie führt zur Senkung des Blutdrucks und wahrscheinlich zu weniger Todesfällen und Transplantat-Verlusten im Vergleich zu Placebo oder Standardbehandlung allein.
- Eine Angiotensinrezeptorblocker (ARB)-Therapie kann den Transplantatverlust verringern.
- Es existieren immer noch zahlreiche Evidenzlücken in diesem Bereich, insbesondere sind RCT („randomized controlled trials“) mit ausreichender Stichprobengröße und längerer Nachbeobachtung erforderlich.
- Die größte Evidenzlücke besteht im Bereich der pädiatrischen Nierentransplantation, hier ist eine differenzierte Betrachtung unabdinglich.
